# Recent evidence-based developments in the treatment of DID

**DOI:** 10.3389/fpsyt.2025.1650164

**Published:** 2025-09-25

**Authors:** N. Bachrach, R. J. C. Huntjens

**Affiliations:** ^1^ Department of Medical and Clinical Psychology, Tilburg University, Tilburg, Netherlands; ^2^ GGZ Oost-Brabant, personality and trauma related disorders, Helmond, Netherlands; ^3^ RINO Zuid, Eindhoven, Netherlands; ^4^ Department of Experimental Psychotherapy and Psychopathology, University of Groningen, Groningen, Netherlands

**Keywords:** dissociative identity disorder, review, treatments for dissociative identity disorder, treatment models for DID, theoretical models for DID

## Abstract

The research field focusing on the etiology, diagnosis and treatment of people with dissociative identity disorder (DID) is still relatively young and limited in scope. Until a few years ago, psychotherapeutic treatment for adults with DID consisted primarily of practice-based, phase-based psychodynamic psychotherapy based, whose treatment effects on dissociative symptoms are small. In recent years, fundamental research on dissociative amnesia and identity functioning has forwarded new insights important for the conceptualization of DID. In light of these emerging insights, empirically supported treatment modalities that have a strong evidence base in adjacent clinical populations have been adapted for application in individuals with DID. Initial results of first empirical studies have indicated positive outcomes, with large effects on dissociative symptoms, of several new treatment options. This review provides an overview of the theoretical models for DID and the foundational research that has led to the development of these models and contributed to adapting treatments with a strong evidence-base in adjacent populations to treat patients with DID. These applications show promising results among individuals with DID. An important next step for the near future is to systematically replicate and extend the evidence base of these promising new approaches in methodologically well-designed and comparative treatment studies. High-quality research is thus urgently needed to identify (cost-)effective treatment options for this population.

## Introduction

Dissociative Identity Disorder (DID), as defined in the Diagnostic and Statistical Manual of Mental Disorders-5 (DSM-5) (1), involves the presence of two or more distinct identity states, accompanied by disruptions in self-perception, memory, and behavior. Individuals often report memory gaps that exceed normal forgetting, particularly regarding daily events or traumatic experiences, causing significant distress or functional impairment (1). The 12-month prevalence of DID is estimated to be 1.5% among American adults (1), rising to approximately 5% in psychiatric settings, with reported rates ranging from 0.4% to 14% in different populations (2). To date, no prospective longitudinal studies have been conducted on the course of DID. Most patients report retrospectively that the initial symptoms of the disorder emerged in early childhood, typically between the ages of 5 and 8 (2). Dissociative symptoms measured by self-report questionnaires appear to occur as frequently in men as in women in the general population. However, dissociative disorders are much more frequently diagnosed in women than in men in clinical practice (3). Compared to individuals with other psychiatric or personality disorders, DID patients experience up to 50% greater impairment (4, 5). They are also at an elevated risk of self-harm, repeated suicide attempts, and mortality, making them among the most costly patients to treat within the healthcare system (6). These clinical features and the high-risk profile underscore the urgent need for developing effective treatment models.

## Theoretical views on DID and controversies

### Theoretical models

The literature broadly distinguishes between three explanatory views for the development of DID: the trauma model (7, 8), the sociocognitive model (9–11), and the schema mode model (12–14).

The *trauma model*, a stress-diathesis model, assumes a direct relationship between early childhood severe trauma (such as chronic maltreatment, abuse and/or neglect) and the development of dissociative symptoms, particularly in individuals with a predisposing (hereditary) predisposition to dissociation (7). Dissociative reactions are seen within this model as a learned coping mechanism for surviving long-term childhood traumatization. This allows a person to cope with the fear and pain and cognitive dissonance arising from these abusive experiences, often caused by people the person depended on as a child (7). A specific form of the trauma model is the *structural dissociation model*. The structural dissociation model, grounded in a trauma framework, proposes that developmental trauma disrupts the child’s capacity to integrate emotional, cognitive, and somatosensory experiences, resulting in a fragmented sense of self; a “structural” division of the personality into multiple compartmentalized (split up) parts as a direct reaction to experiences of trauma and milder stressors. These parts would also be characterized by dissociative amnesia: Gaps in memory for what occurred when another identity took control of behavior (15). According to this model, emotionally vulnerable parts—referred to as “emotional personalities”—have memories of traumatic experiences as experiences and become fixed in the past, retaining the affective states and survival mechanisms linked to the traumatic experiences. Subtypes of emotional personalities reflect different survival strategies such as fight, flight, and freeze. In contrast, “apparently normal personalities” remain unaware (i.e., amnesic) of the traumatic events due to psychological compartmentalization. These personalities take care of responsibilities in daily life and subtypes may focus on, for example, work or taking care of children. Over time, the patient may develop more personalities to deal with other types of negative events, including milder stressors, and daily responsibilities (16). To support this model, neurobiological research has been presented claiming to have identified a biomarker for dissociative amnesia (17).However, this research has been criticized, for example because as an index of dissociative amnesia, only scores on subjective self-report measures were used instead of scores on cognitive memory tasks and it was not clear if the results were specific to DID or also evident for other comorbid disorders (18). Moreover, recent reviews of neuroscientific research on dissociative symptoms concluded that no reliable biological markers have been identified, and the brain regions implicated vary considerably across studies (19). For example, a review by Lotfinia and colleagues (20) of 33 articles studying patients reporting dissociative symptoms indicated that dissociative processing cannot be localized to a few distinctive brain regions (also see 19). Finally, it is important to mention that the reversible nature of dissociative amnesia seems incompatible with structural brain abnormalities. Alternative explanations, such as dysfunctional beliefs about memory, may provide a more plausible for reported amnesia in DID.

The *sociocognitive model* explains dissociative symptoms as arising from social, cognitive and cultural causes. This model does not assume a direct relationship between dissociation and childhood trauma. Instead, it assumes that patients come to believe they are made up of multiple parts under the influence of information in the media (including series, websites, movies and books), sociocultural beliefs (in which DID is considered an accepted way of expressing mental health problems) and suggestive techniques from practitioners. The model does not deny that patients suffer from valid psychological complaints. However, it argues that, in the search of an explanation of the current complaints, the practitioner may suggest a traumatic past, may interpret mental images as evidence of repressed memories or suggest the patient suffers from dissociative amnesia if she/he has no images, and/or may interpret for example different emotional states as identities. Patients and practitioners are seeking an explanation for emotional instability, identity problems, and impulsive behavior, characteristics frequently present in borderline patients, among others (21, 22). This model is sometimes erroneously called the fantasy model by proponents of the trauma model, a somewhat disparaging designation since this term reflects neither the core nor the scope of the model. From this perspective a potential issue within this target group is the development of pseudo-memories. Psychological treatment can lead to both the increased occurrence of genuine memories and the emergence of fictional memories due to the use of suggestive treatment techniques and misconceptions by patients and therapists about how memory functions. One example of such a misconception is the belief that traumatic memories can be “repressed” and must be retrieved for healing to occur. As a result, emerging images may be incorrectly interpreted as memories. Furthermore, images of (sexual) abuse may be a mix of actual events, fantasies, reconstructions, or the merging of different event (e.g., 23, 24). If a patient does not initially report abuse or other trauma, therapists should avoid suggesting the possibility of traumatic experiences.

In newer formulations of the above models, similarities can also be found between them (8, 10). For example, both models now assume that multiple factors can contribute to the development of dissociative symptoms such as genetic predisposition, dysfunctional family relationships, negative experiences in childhood and lack of social support. Moreover, newer formulations of the sociocognitive model include the possibility that people with DID have experienced trauma in childhood, although a direct causal role is not assumed (10).

### New insights in the conceptualization of DID

In addition to the theoretical developments described, findings from fundamental empirical research on inter-identity amnesia and further cognitive identity functioning have forwarded new insights important for the conceptualization and treatment of DID. Contrary to the assumption of the presence of a structural” division of the personality” into multiple compartmentalized (split up) parts among DID patients, research of the past 20 years in which objective tasks were used to measure inter-identity amnesia has repeatedly shown that this assumption is incorrect. Transfer of knowledge between identities was found in terms of episodic, semantic, autobiographical and for example also procedural memory, and both for neutral as well as self-relevant and trauma-related information (see e.g. 25–31). Additionally, it has been shown that DID is related to impairments in subjective self-clarity, but not to differences in the self-concept structure when more indirect, objective cognitive tasks are used (32). An alternative explanation for the experienced inter-identity amnesia and compartmentalization between personality states that does align with these empirical findings assumes dysfunctional metacognitive beliefs about memory and the self. These beliefs might keep patients, despite an intact memory system, from retrieving trauma-related memories on subjective memory tasks (e.g., tasks on which they are simply asked to retrieve what they have learned in another identity state) because of fear of losing control or “going crazy”. Other examples include the belief that it is better to forget the painful events that you have experienced in your life (33).

A second important line of research that has contributed to new insights in the conceptualization and treatment of DID relate to the timing of trauma focused treatment for patients with early childhood trauma, such as DID patients. More specifically, studies in people with PTSD as a result of early childhood trauma have shown that adding a stabilization phase prior to trauma processing phase (aimed at symptom reduction and usually consisting of emotion regulation, safety planning, and coping skills training prior to trauma processing) is not necessary and has no added value for the treatment of the majority of people with PTSD symptoms due to early childhood trauma. These studies found large effect sizes for reducing PTSD symptoms as well as other symptoms such as depression, dissociative symptoms, and trauma related cognitions with trauma-focused treatments in complex PTSD patients, with low dropout rates and low rates of serious adverse events (34–37). In addition, recent studies have showed that trauma-focused therapy for PTSD in people with (somatoform) dissociation is also effective (38; 39).

Thirdly, various studies have shown that patients with dissociative disorders have high levels of comorbid personality disorders, especially avoidant, borderline, dependent and schizotypal features (40, 41). Patients with DID can be characterized by personality disorder traits related to avoidance of social contact due to a fear of being criticized or rejected and due to feelings of insufficiency and inferiority as well as a high fear of disconnection and rejection whereby DID patients often find it very difficult or are even unable to form secure, satisfying attachments to others. They feel essentially different, insufficient, and disconnected from others (42). DID patients are characterized by high levels of avoidant coping strategies (i.e., avoidance of internal or external trauma-related information) (12). Dysfunctional coping strategies have been found to significantly predict dissociative symptoms and mediate the relationship between trauma exposure and dissociation, highlighting the potential role of coping processes in the etiology and maintenance of dissociative phenomena (43). These data raise the question of whether DID should be viewed as a separate diagnostic category with its own unique treatment model. Or whether the overlap with other disorders is so great that we can apply effective treatments used in adjacent disorders to this group. These empirical findings have opened the way to the development of new theoretical models explaining DID, such as the schema mode model of DID.

### Recent new theoretical model

A recent theoretical model, developed in part based on the empirical research mentioned above, is the schema mode model for dissociative disorders (12–14). According to the original schema mode model, developed by Young et al (44), psychopathology arises through the interaction between genetic vulnerability, temperament, and environmental factors such as childhood neglect or traumatization (44). Negative experiences and frustration of basic needs in childhood (such as safety, connectedness, and autonomy) lead to the development of early maladaptive schemas. A schema consists of structures of coherent knowledge elements about the person himself, about the world, and about the person’s interaction with the outside world. For example, abuse experiences in early childhood can lead to the development of a schema in which mistrust/abuse is central. In such a schema, people have the expectation that they will be used, treated badly, humiliated or abused by others. In addition to schemas, people also develop modes early in life. These are moment-to-moment emotional, cognitive, and behavioral states (44) that are the result of how individuals cope (resignation, avoidance or inversion) with an activated schema. ST describes several categories of maladaptive modes: dysfunctional child modes, dysfunctional coping modes, dysfunctional critical and/or punitive modes and healthy modes.

The schema mode model for dissociative disorders entails both aspects of the trauma model and the sociocognitive model but explains dissociative symptoms through modes and shifts between modes. Each person has modes, both healthy individuals and individuals with psychopathological symptoms, rendering the model dimensional and normalizing the experience of different cognitive-emotional-behavioral states. Some modes, however, are more prevalent in individuals with psychopathology. In addition, these individuals are characterized by more frequent shifts between modes or rigidity in the modes. Mode shifts can occur smoothly and gradually but can be more abrupt and extreme in individuals suffering from severe psychopathology such as DID. This assumption is supported by a recent study which found that scores of individuals with DID on maladaptive personality traits and schemas were comparable to the scores of individuals with borderline personality disorder and avoidant personality disorder (42). Common modes in people with dissociative disorders include the vulnerable child, critical modes, and various avoidant coping modes (42). Following the empirical research that showed that identities in people with DID are not as compartmentalized as previously assumed (i.e., given transfer of information between identities), the schema mode model does not assume compartmentalized identities. In ST for DID, the subjectively experienced identities as well as the experienced inter-identity amnesia reported by individuals with DID is validated and acknowledged, however, they are understood as the result of dysfunctional metacognitions driven by avoidance of internal and external trauma-related stimuli (45). At the same time, this model guards against reification, that is, labeling identity states as separate “persons”. The personality states are categorized by function and underlying need in consultation with the patient and then re-labeled as modes. There is a focus in this model on both the consequences of negative experiences in childhood and the effects they may have had on personality and identity development and comorbid symptoms.

### Treatments for DID

Evidence-based guidelines for the treatment of dissociative disorders are still not available due to the lack of solid empirical data. Until a few years ago, psychotherapeutic treatment for adults with DID consisted primarily of practice-based, phase-based psychodynamic psychotherapy based on the trauma model (46). The above mentioned empirical findings have helped to substantiate the adaptation and application of various evidence-based treatments that have been found to be highly effective in adjacent disorders, such as (complex) PTSD, emotional disorders and personality disorders. These treatment models include Unified Protocol (UP), schema therapy (ST), Cognitive Behavioral Therapy (CBT), Dialectical Behavioral Therapy (DBT), and Mentalization-Based Treatment (MBT). The application of these treatment models within this patient group and its evidence base will be discussed below. **Table 1** gives an overview of the evidence base of these treatments. Before we move on to describe the newer approaches, first we will describe the often applied practice-based approach phase-oriented psychodynamic psychotherapy and the evidence for it, which departs from the structural dissociation model, despite empirical evidence challenging the assumptions of this model.

**Table 1:** overview of the evidence base of treatments for DID.

Treatment	Study	N and sample	Study Design	Outcome
Phase-oriented psychodynamic psychotherapy	Jepsen et al. (47)	N=23, combined group of DID/OSDD	Naturalistic	Small effect for dissociative symptoms (Cohen’s *d* = 0.09) after three months, and at follow-up after 12 months (*d* = 0.25)^*^.
	Brand et al. (48) , 49)	N = 226, Combined group of DID/OSDD	Longitudinal naturalistic	Small effect for dissociative symptoms after 30 months (*d* = 0.3)^**^.
	Baekkelund et al. (50)	N= 59, Combined group of DID/OSDD	RCT	No improvements in dissociative symptoms pre – follow up (*d* = -0.1 in stabilization group, and group without stabilization (*d* = 0.2)^*^.
	Brand et al. (51)	N=291, DID (57.39%), dissociative subtype PTSD (14.78%), OSDD (13.06%) complex PTSD (11%), DDU (3.78%)	RCT	Small effect for dissociative symptoms after 12 months, entire group (*d* = 0.38)^**^
Unified Protocol	Mohajerin et al. (52)	N=5 DID	Repeated-case series	All five patients did no longer meet criteria of DID, large effect for dissociative symptoms 6-month follow up (unweighted average effect size Hedges *g* = 2.08)^*^
Schema therapy	Huntjens et al., in preparation (53)	N= 10 DID	Multiple baseline repeated-case series	6 out of 8 patients no longer met criteria for DID at follow up, large effect on dissociative symptoms (Cohen’s *d* = 1.49)^*^
CBT	54	N=1 DID	Case report	Patient did no longer meet DSM-5 criteria for PTSD and DID, large effect for dissociative symptoms, intake to 6 months follow up (*d* = 2.82) ^***^
DBT	55	N=1 DID	Case report	Authors report reductions in self-harm and suicidal behavior and dissociative symptoms, no objective measurements reported.
MBT	n.a		No study performed on DID	

^*^ Effect size as reported in article.

^**^ Cohen’s d was calculated by calculating the mean difference between the two groups, and then dividing the result by the *pooled* standard deviation.

^***^ Cohens D calculated (within-subject effect size) by taking the difference between intake and follow-up, divided by the SD of the baseline measure distribution in the relevant population.

DDU, dissociative disorder, unspecified.

Different Cohen’s d formulas might be used in studies, making direct comparison between studies difficult.

### Phase-oriented psychodynamic psychotherapy

Phase-oriented psychodynamic psychotherapy is based on the trauma model and is conducted in three phases (46). The first phase focuses on establishing safety, stabilization, and symptom reduction, creating a therapeutic relationship, promoting internal cooperation between identities and learning emotion regulation skills. This phase aims to improve the patient’s ability to emotion regulation and cope with the tasks of daily life, in order to then benefit from trauma processing (in phase 2). The ISSTD practice-based guideline (2011) states contra-indications for moving to the trauma processing phase. Trauma processing should be delayed if there is chronic low functioning, severe attachment problems, minimal ego strength or coping skills, substance dependence, ongoing abuse, medical problems, or severe personality problems. Because such patient characteristics are frequent in DID, for a long time the percentage of patients who progressed to the second stage (and third) stage was relatively low (17-33%, 56; 57). In recent years, the transition to the second phase of treatment is more rapid and the phases are used less statically. Stabilization and trauma processing are more interspersed. In the second phase, processing and integration of the traumatic memories and cognitive restructuring takes place. The trauma processing techniques used include “guided synthesis.” In this technique, identities are required to share their traumatic experiences in a stepwise and controlled manner with other identities that indicate amnesia to the traumatic experiences. In addition, modified forms of trauma processing such as Narrative Exposure Therapy (NET) (58) and a adapted form of EMDR have also been used in recent years (e.g., 59). Guided synthesis and the adapted form of EMDR as well as NET have not been empirically studied thoroughly among DID patients. The third phase focuses on personality integration and recovery of social functioning. Integration is not always desirable or feasible; a stable, coherent inner world may then be sought. There is also a focus in the third phase on grief, therapy completion, relapse prevention and the future. On average patients with DID were approximately 8.4 years in treatment with their current therapist in the final phase of their treatment in a naturalistic study among community clinicians. It should be noted that the treatment had not yet been completed and some cases extended up to 20 years of treatment (48), and dropout can be high (e.g., 60% in a study by 57; 68% in 60).

The effectiveness of phase-based treatment has been examined in a number of non-controlled studies. Jepsen et al (47) followed 23 inpatients (DID/Other Specified Dissociative Disorder (OSDD)) who received a 3-month treatment aimed at stabilization (phase 1). There was virtually no effect on dissociative symptoms (Cohen’s *d* = 0.09) after three months, and a small effect (Cohen’s *d* = 0.25) at follow-up measurement 12 months. The largest study to date, a study by Brand et al (48), 49), compared a large group of patients (N = 226 DID/OSDD) at different stages of treatment and followed the entire group for 30 months over time. The therapists in this study were positive about their patients’ progress on overall functioning, psychosocial functioning and self-harm. Patient scores were lower but also positive. Patients did report improvements on PTSD symptoms, general psychopathology complaints and self-harm. After 30 months, patients reported a small improvement in dissociative symptoms (a mean of 28.8 on the DES after 30 months compared with a mean score of 35.4 at baseline, Cohen’s *d* = 0.3). Patients in the final phase of treatment reported no significant improvements on dissociative symptoms, depressive symptoms and number of admissions, alcohol and drug use, voluntary and paid employment and suicide attempts. There was no control condition or waiting list control groups in this study and no treatment protocol which limits generalizability. As a result, it cannot be ruled out that the improvement in functioning was due to natural recovery.

In a first Randomized Controlled Trial (RCT) in this field (50), 59 patients with DID/OSDD were randomized into two conditions, with either stabilization group treatment of 20 sessions in addition to individual therapy versus individual therapy without stabilization group. Group treatment was based on the book “Coping with Trauma-Related Dissociation” by Boon et al (61). Patient reported improved psychosocial functioning significantly better in the condition with additional stabilization group (Cohen’s d = .87) than in the condition without stabilization group (Cohen’s d = .65). However, in both conditions no improvements were apparent on dissociative symptoms (Cohen’s *d* = -0.1 stabilization group, *d* = 0.2 without stabilization group). It was surprising that patients in the condition without the stabilization program showed significant progress on PTSD symptoms (Cohen’s *d* = 0.6 as well as on other psychological symptoms (Cohen’s *d* = .4), while this was not the case in the stabilization condition (Cohen’s *d* = -.1 PTSD and *d* = 0.1 for psychological symptoms). The dropout rate was 23%. The results thus indicated that participation in stabilizing group treatment does not lead to reduced scores on the core symptoms (i.e., dissociative symptoms) for patients with complex dissociative disorders, at least not treatment based on the book of Boon et al. (61). The authors emphasized that there is much room for improvement in the treatment of patients suffering from complex dissociative disorders such as DID and OSDD. The interpretation of the above results is complicated by the fact that no information was collected on the content of individual treatment. It is possible that therapists in the stabilization condition were not yet provided trauma, which could explain the improvement in PTSD symptoms in that condition. However, it is also possible that the difference between the groups on PTSD symptoms is due to potentially adverse effects of a stabilization phase: after all, participants in this condition get the message “not yet ready” for trauma treatment, which can lead to a decrease in self-efficacy and autonomy (62).

A recent RCT of Brand et al (51) investigated the effectiveness of Finding Solid Ground (FSG), an online psychoeducational program, as an adjunct to ongoing psychotherapy for individuals with trauma-related dissociation (TRD), including patients with dissociative identity disorder (DID), the dissociative subtype of PTSD, and complex PTSD. A total of 291 international outpatients were randomly assigned to either immediate access to FSG or a 6-month waitlist control group. Participants continued to receive regular outpatient psychotherapy, and no exclusions were made for suicidality, non-suicidal self-injury, recent hospitalization, or substance use, enhancing the ecological validity of the study.

The interim report of the study results showed that after 6 months of access to the FSG program, the Immediate group showed moderate improvements with effect sizes of g = 0.72 for emotion regulation, g = 0.57 for PTSD symptoms, 0.69 for self-compassion, and 0.58 adaptive functioning for compared to baseline. These improvements increased to large effect sizes after 12 months, with g = 1.32 for emotion regulation, g =1.20 for PTSD symptoms, g = 0.98 for self-compassion, and g = 0.95 for adaptive functioning. At the 6-month follow-up, the Immediate FSG group showed significantly greater improvements than the Waitlist group in key outcomes such as emotion regulation, PTSD symptoms, self-compassion, and adaptive functioning. However, with regard to core DID symptoms, the results indicated that dissociative symptoms changed significantly over time, albeit with a small effect size (*d* = 0.38) across all participants in the interim sample. However, there were no significant differences between the treatment and control groups, nor was there a significant interaction between group and time, which suggests that while dissociation levels fluctuated during the study period, these changes were not specifically linked to the intervention. Interpreting the above results is challenging because data on the specific content of each individual’s treatment were not collected. The exclusive use of self-report measures raises concerns about possible response bias, semi structured clinical interviews might performed by independent research assistants might prevent such biases. Additionally, the intervention was delivered alongside unstandardized psychotherapy, complicating causal interpretations.

## Adaptations of treatments with an evidence-base in adjacent disorders

### Unified Protocol

The Unified Protocol (UP) for DID is a transdiagnostic treatment for patients with emotional disorders (63). It is a short-term, protocol-based treatment aimed at teaching coping strategies and skills to better regulate emotions and focusing on awareness of thoughts, feelings, and behaviors and developing more cognitive and behavioral flexibility. The protocol consists of 18–22 sessions and has a set structure: psychoeducation and rationale for treatment, increasing motivation, emotional awareness, cognitive restructuring, reducing emotional avoidance, awareness of physical sensations, imaginary exposure to traumatic memories, exposure *in vivo*, and relapse prevention (52). Improving sleep is also part of UP treatment for patients with DID/OSDD, as sleep problems can exacerbate dissociative symptoms (64). These interventions focus on sleep improvement through psychoeducation, sleep hygiene, sleep restriction, mindfulness, positive imagery, and relaxation techniques. The UP protocol is used in DID patients in a more flexible manner (e.g. repeating particular session, change of order of sessions), especially in the case where identities interfere with a standard implementation of the UP treatment protocol (52).

The UP model aligns with the sociocognitive model that assumes that DID/OSDD arises because vulnerable patients have come to understand their behaviors, feelings and cognitive problems as multiple identities. Therefore, the intervention does not aim to integrate individual personality states, but focuses primarily on learning how to manage emotions. There is recognition that the patient experiences these personality states, but the sense of separate identities is not reinforced and the personality states are not reified (i.e., an intangible concept is treated as a tangible entity) into separate persons (52).

The effectiveness of UP has been studied for various emotional disorders. A recent meta-analysis showed large effect sizes (baseline to follow-up) for patients with anxiety and depression. UP outperformed wait-list control and treatment-as-usual; compared with traditional CBT for emotional disorders, the effectiveness of UP ranged from equivalent to modestly better (65). In DID, the UP protocol was examined in a repeated-case series with five patients (52). After 18–22 sessions, 4 patients showed significant reductions in anxiety, depression and dissociative symptoms, and an increase in emotion regulation. These improvements were maintained at follow-up at 1, 3 and 6 months. A fifth participant with prominent suicidal ideations was treated for 42 sessions and achieved similar improvement in symptoms. All five patients did not longer met criteria for DID or any other disorder at 6-month follow-up, large effects were found for dissociative symptoms at 6-month follow up (unweighted average effect size Hedges *g* = 2.08)^*^. Although patients continued to experience different personality states, they identified these as different aspects of themselves. Patients also reported that dominating personality states could be effectively controlled with mindfulness techniques.

### Schema therapy

Schema Therapy (ST) for Dissociative Identity Disorder (DID) consists of two weekly sessions during the first two years, followed by one weekly session in the third year and six monthly booster sessions, totaling 222 sessions of approximately 50 minutes each. The treatment is based on the ST model by Young et al. (44) and adapted to address the complex needs of DID patients. A key aspect of ST for DID is validating patients’ subjective experiences of identity shifts, fluctuating senses of agency, and inter-identity amnesia, without assuming fixed compartmentalization. Through psychoeducation, identity states are gradually reformulated as schema modes, forming the basis for a mode-based case conceptualization and intervention plan. ST offers both patients and therapists concrete tools to deal with these shifts, by increasing awareness of the modes, followed by learning strategies for dealing with the (shifts between) modes as well as learning to handle and change the modes. Through limited reparenting in the therapeutic relationship (i.e., attachment relationship within which the therapist offers what a person missed in his or her childhood) and ST techniques specifically adapted for DID such as the multi-chair technique, imaginary rescripting and (historical) role-playing and cognitive and behavioral therapeutic interventions.

Avoidance—both behavioral and cognitive—is a central feature of DID. ST addresses this through empathic confrontation and experiential techniques (e.g., chair work, imagery, exposure), guided by understanding which modes drive the avoidance. The Detached Protector mode, characterized by emotional withdrawal and depersonalization, is common in DID and seen as a response to chronic stress. Addressing this mode helps foster emotional reconnection and therapeutic engagement. Punitive and demanding modes, often echoing past abuse, are deeply ingrained and harmful. Though initially experienced as integral, these parts are gradually challenged and eliminated using techniques such as imagery, rituals, and chair work. This process requires persistence from both therapist and patient. Trauma processing is conducted via Imagery Rescripting (ImRs), starting with psychoeducation and progressing from mild to more severe memories as tolerated. Patients move from observing to actively participating. Therapist start processing trauma early in treatment quickly and do not use a phase treatment approach. While the standard protocol is followed, adaptations are often needed due to avoidance, punitive parts, and self-harming tendencies. Promoting autonomy is critical, as DID patients often feel powerless and dependent. ST supports autonomy through structured goal setting, home practice, and reducing reliance on caregivers. Therapists also help patients shift from avoidance-based to approach-oriented goals and foster identity development. Given the chronicity and hopelessness common in DID, regularly reviewing strengths and progress is essential. Emphasizing therapeutic gains using tools like mode pie charts helps maintain motivation and supports recovery.

ST, as well as its trauma processing component, Imagery Rescripting (without stabilization phase) have been shown in several randomized studies to be effective and safe, with large effect sizes, for disorders related to interpersonal trauma in childhood, including complex PTSD and severe personality disorder and dissociative symptoms in BPD patients (e.g., 34, 36, 66–69). The effectiveness of the modified form of ST for DID is currently being investigated in two case series design studies. A first study involved 10 patients with DID and a second study 32 (12–14). Case reports appeared on two of the participating patients from the first study describing modified ST treatment. The results of the first study (12), are promising as 6 out of 8 patients no longer met criteria for DID at follow up, large effects were found for dissociative symptoms at follow up (Cohen’s *d* = 1.49) (Huntjens et al., in preparation).

### Cognitive behavioral therapy consisting of direct trauma-focused intervention and “saying goodbye” to identities

The CBT model for DID consists of brief intensive trauma-focused treatment involving trauma processing combined with “saying goodbye” to identities. The intensive trauma treatment consists of Imaginal Exposure combined with EMDR. During this treatment, avoidance behaviors are actively broken and dysfunctional meta-memory beliefs that perpetuate pathology are debunked. During (imaginal) exposure, patients are motivated to approach the feared stimuli, both traumatic memories and avoided situations or objects, without safety behaviors. As a result, patients learn that their dysfunctional metacognitive beliefs (e.g., “if I remember the details of my trauma, I will lose control”) are falsified (42, 54).

Dissociation in this view is not seen as an “automatic” reaction over which one has no control, but as an avoidant coping strategy, that one can gain control over, in order to avoid being overwhelmed by strong emotions. Patients are encouraged to take active control of their dissociative coping responses and confront the traumatic memories. Symptoms of dissociation, such as depersonalization (e.g., “I don’t feel my body”), somatoform dissociation (e.g., paralyzed legs), experienced amnesia (e.g., “I can’t access the trauma memory”), and identity fragmentation (e.g., another identity pops up) are consistently labeled as avoidance behaviors (e.g., “You are dissociating now to avoid the traumatic memories, try to let go of this avoidance behavior and fully expose yourself to the traumatic memory”). The therapist does not intervene in dissociative episodes, by providing relaxation or grounding exercises, but encourages the patient to overcome avoidance and approach the traumatic stimuli themselves. In addition, dysfunctional beliefs about memory and dissociation are actively countered (“I can’t remember” is relabeled to “You think you can’t remember, but you probably mean: ‘I’m too afraid to remember because I will be overwhelmed and lose control’). Within this model, it is assumed that by decreasing PTSD symptoms, dissociative symptoms no longer have a function, and will also decrease alongside with the PTSD symptoms (54).

Because many DID patients have spent years “living” with their identities, which can sometimes feel like “imaginary friends,” additional sessions are spent on imaginary “saying goodbye” to their identities. This is done using a farewell ritual. Identities are seen in this model as survival strategies to cope with unpleasant and drastic events. These used to be functional but are no longer helpful in the present and so they can be parted with. Together, therapist and patient explore which identities the patient wants to say goodbye to. Then, as a first step, they analyze the function of each of the identities. In the next step, the patient is imaginarily exposed to an identity, after which the identity is thanked for the function the identity had during the traumatic experiences. Next, patient indicates towards the identities that the patient can handle life on his or her own and no longer needs the identity, after which the identity is imaginary waved goodbye (54).

CBT for DID builds on the proven effectiveness of intensive trauma treatment for disorders resulting from interpersonal trauma in childhood. Voorendonk et al. (70) showed in a non-randomized study the effectiveness of an intensive 8-day treatment consisting of a combination of exposure, EMDR therapy, psychoeducation and physical activity, for complex PTSD. Both PTSD and complex PTSD decreased significantly during treatment in 308 patients diagnosed with PTSD. This resulted in a significant loss of PTSD (74.0%) and complex PTSD diagnoses (85.0%) No side effects occurred in terms of suicides, suicide attempts, or hospitalizations. No RCTs have yet been conducted on the application of this intervention in the DID/OSDD patient group. In a case study, 54) describe the application of CBT for DID. Treatment outcomes were measured at intake, before treatment, after treatment, and at 3 and 6 months of follow-up. After treatment, patient no longer met DSM-5 diagnostic criteria for neither PTSD nor DID. These outcomes were maintained at follow-up as large effects were found for dissociative symptoms at 6 months follow up (Cohens *d* = 2.82).

### Dialectical behavior therapy

Dialectical behavior therapy (DBT) is an evidence based treatment developed specifically for chronically suicidal, self-harming and severely BPD patients (71, 72). DBT is a behavioral treatment based on a biosocial theory of personality, mindfulness and dialectics. Central goal of DBT is to improve emotion regulation and self-acceptance. Dysfunctional emotional regulation is viewed in this model as the result of an interaction between biologically emotionally vulnerable individuals and a disabling environment, which communicates to the person that their behavioral, emotional and cognitive responses are inadequate. Due to disability, patients do not learn to recognize, label and modulate (intense) emotional experiences and do not develop confidence in their own experiences. As a result, patients experience themselves as a vessel of contradictory, changeable and intense emotions and states (71). This, in turn, complicates the formation of supportive long-term relationships. Dissociation is understood primarily as a coping mechanism that arises in response to overwhelming emotional distress. DBT explains dissociation as a way in which individuals protect themselves from intense emotions or painful memories by disconnecting from their present experience, which can interfere with effective emotional regulation and engagement in therapy. DBT, focuses on helping clients develop mindfulness and distress tolerance skills to reduce dissociation and improve their ability to stay present and manage difficult emotions more adaptively. Recently, DBT-PTSD has been specifically developed to address the unique needs of individuals with Complex PTSD (73). It is grounded in the core principles of dialectical behavior therapy (DBT) and incorporates prolonged exposure with DBT. A randomized controlled trial assessing a residential DBT-PTSD program showed a significant reduction in posttraumatic symptoms, with large effect sizes compared to a waitlist control group receiving standard care (Cohen’s d = 1.5). Early childhood traumatization is considered a major cause of DID within the DBT model, but it does not assume structural dissociation of personality. Therefore, DBT does not aim to integrate individual personality states, but the goal is to reduce therapy-threatening behaviors such as dissociation, self-destructive or suicidal behavior (55).

DBT consists of weekly individual outpatient DBT sessions and weekly DBT group skills training for one year. In individual and group training, patients learn psychosocial skills (including emotion regulation, stress tolerance, impulse control, interpersonal effectiveness, coping with crisis and mindfulness). DBT recommends a staged treatment approach with regard to trauma processing, which begins with establishing safety and stabilizing symptoms before addressing traumatic material. In DBT, this corresponds to Stage 1, focusing on behavioral control and safety, while trauma processing is reserved for Stage 2.The main rationale for adapting and applying DBT in DID is the significant overlap between BPD and DID, in terms of self-harm and suicidality in both disorders and the strong comorbidity between DID and BPD (3). Given these similarities, the DBT model can be applied to DID without major modifications. However, the clinician will also often encounter clinical features of DID that require modifications to the DBT approach. Foote and van Orden (55) suggest the following three principles regarding these adaptations:

1.Since DID patients are quite similar to BPD patients in terms of the frequency of para-suicidal behavior, treatment first focuses on stabilization of these symptoms (phase 1) prior to trauma treatment (phase 2).
In DID patients, frequent dissociative behaviors will be present, such as various personality states that may cause problems (including impulsive acting or self-injury). Often the patient reports amnesia for these behaviors, which makes the therapeutic process difficult. Therefore, dissociative behaviors that disrupt therapy or are life-threatening should be treated in Phase 1.
3.DBT does not necessarily view switching between states as maladaptive. The functions and consequences of these behaviors are analyzed first. If there is a maladaptive pattern of switching behavior, treatment will focus on reducing that specific switching behavior.

DBT has been shown to be effective (large effect) in multiple RCTs for the treatment of patients with BPD. In BPD with comorbid PTSD and severe self-injury problems, standard DBT extended with exposure for trauma processing appears to be effective and safe (74). No RCTs have been conducted on application of this intervention in DID patients. In a case report, Foote and Van Orden (55) observed reductions in self-harm and suicidal behavior and dissociative symptoms, in a DID patient treated with DBT for 2 years. However, the reported changes were not substantiated through (semi)structured interviews or patient reporting.

### Mentalization-based treatment

Mentalization-based treatment (MBT) was developed for adult BPD patients (75). The goal of MBT is to learn to “mentalize. Mentalization is the ability of people to understand themselves and each other by learning to understand and explain their own behavior and that of others from underlying feelings, thoughts, desires and intentions. The ability to mentalize develops during childhood within a secure attachment context, when children within a secure attachment relationship feel themselves adequately mentalized in the mind of the educator (75). In MBT, mentalizing ability is believed to be underdeveloped in BPD patients due to genetic vulnerability interacting with traumatic (attachment) experiences, resulting in hyper- and hypoactivation of the attachment system (75). A basic premise in MBT is that in patients, when tension mounts, especially in the context of close relationships, the ability to mentalize is rapidly lost and recovers with difficulty, manifesting as feeling quickly overwhelmed, feeling empty, filling in thoughts for others, feeling quickly rejected or criticized. At such times, they switch to pre-mentalizing modes (75). In these non-mentalizing modes, the person often experiences and functions in a primitive way in relation to psychological reality. Three pre-mentalizing modes are defined in MBT: the psychic equivalence mode (what I think is reality), the as if mode (in which there is precisely no longer any relationship between what the patient thinks and feels and reality) and the teleological mode (in which only goal-directed actions exist). In addition, patients often experience an incoherent and fragmented sense of self. Feelings are often so intolerable that the only way to stabilize the self lies in destructive behavior or externalizing behavior (75). The MBT literature does not explicitly describe a separate explanatory model for DID. Within MBT, traumatic experiences are seen as a transdiagnostic vulnerability factor of psychopathology. Dissociation is considered a primitive form of mentalizing, i.e., an expression of the as if mode, a state in which the inner and outer worlds are kept separate. Relapses are seen as an expression of the psychic equivalence mode. The psychic equivalence mode refers to a mental state in which an individual experiences their internal thoughts, feelings, and perceptions as directly and unambiguously reflecting external reality. In this mode, there is a collapse of mentalization—the ability to understand that one’s own mind and others’ minds are separate and subjective. As a result, the person treats their internal experiences as absolutely true, leading to rigid and concrete thinking (75).

Within MBT, current mental states are worked with in the here and now. To the extent that the past is invoked, therapy focuses on the role of past experiences and how these experiences may have affected the present state. Trauma treatment within MBT therefore consists of mentalizing the traumatic experience and breaking through re-enactment, which causes patients to continually re-experience the past in the present. More recently, a more specialized treatment program has been develop specifically for patients suffering from attachment or complex trauma: Trauma-Focused mentalization-based treatment, which is an adaptation of MBT in which specific interventions are used to increase mentalizing capacities by directly addressing the impact of trauma (76). MBT consists of one or two group psychotherapies per week, combined with individual psychotherapy for at least one to one and a half years and is also offered in more intensive variants in the form of two-, three- and five-day intensive part-time treatment.

MBT is an evidence-based treatment for patients with (severe) BPD and results in significant reductions in BPD traits, self-harm, suicidality, general psychiatric symptoms, improvement in general functioning and social functioning (77–81). MBT appears to be more effective than traditional psychodynamic group therapy as the severity of BPD increases (82). MBT has not been studied in patients with DID/OSDD. Within some treatment centers, patients with DID/OSDD are admitted to MBT treatment programs (83).

## Psychopharmacology

Patients with DID are frequently prescribed medications to address the complex range of co-occurring, non-dissociative psychiatric symptoms that commonly accompany the disorder (84). It should be noted, however, that no pharmacological trials targeting pathological dissociation in DID have been carried out to date and therefore need to be empirically investigated (84). In individuals with adjacent disorders, the effects of two classes of medication on dissociative symptoms — paroxetine and opioid antagonists— have been investigated (84). One RCT demonstrated significant reduction of dissociative symptoms, measured with the DES, compared to placebo in PTSD using a selective serotonin reuptake inhibitor (SSRI), paroxetine (85). Another randomized controlled trial showed that the SSRI fluoxetine did not perform better than placebo in reducing dissociative symptoms in individuals with depersonalization disorder (86).The evidence remains for opioid antagonists as potential treatments for dissociative symptoms also is scarce and inconsistent. Initial findings, such as naloxone’s reduction of depersonalization symptoms in a single-blind trial (87) and supportive case series with naltrexone and nalmefene in patients with depersonalization disorder (86), borderline personality disorder (88), and PTSD (89), patients with severe trauma-related and dissociative disorders (90), suggest possible benefit. However, these results have not been replicated in more rigorous study designs. In fact, small double blind randomized controlled trials among individuals with borderline personality disorder found no superiority of naloxone or naltrexone over placebo in reduction of dissociative symptoms (91, 92), and naloxone did not block ketamine-induced dissociation in a double-blind crossover study (93). Taken together, the existing evidence base is methodologically weak and inconsistent, and it remains uncertain whether opioid antagonists hold clinical utility for dissociative symptoms, let alone for patients with DID, for whom systematic trials are entirely lacking.

## Discussion

Research on treating dissociative disorders is limited despite the significant suffering involved (6). DID patients are often excluded from larger (epidemiological) studies and given the length of treatment, RCTs have not been regularly conducted. Excluding DID patients from epidemiological studies perpetuates under-recognition, leaves major gaps in the knowledge about the prevalence and course of DID. The exclusion of DID patients in clinical trials contributes to a lack of evidence-based care for this group, stigma and inequities in treatment access. Inclusion of DID patients in clinical trials can be improved through the use of validated diagnostic interviews for DID, explicit eligibility criteria for DID patients, and preplanned subgroup analyses. Multi-site consortia incentives for this subgroup can address low prevalence rates of DID. Increased funding opportunities can stimulate research in this field and finally involvement of individuals with lived experience can enhance feasibility and recruitment.

Traditionally, adult DID treatment followed a phase-oriented psychodynamic trauma model (46), studies investigating the effectiveness of phase-oriented psychodynamic treatment for DID, show small treatment effects on dissociative symptoms. Recent research challenges the idea of “structurally divided” identities, suggesting that inter-identity amnesia results from dysfunctional beliefs about memory and trauma rather than actual memory transfer deficits. This shift views DID as a disorder of self-understanding, focusing on mistaken beliefs about memory functioning and identity fragmentation. Trauma-focused treatments without prior stabilization have shown effectiveness for DID symptoms and related clinical groups. Additionally, DID patients often exhibit comorbid personality traits, such as avoidant and borderline features, leading to attachment difficulties (42). These findings support adapting evidence-based trauma treatments and therapies like CBT, DBT, and ST, originally developed for PTSD and personality disorders, to treat DID. Initial results of first empirical studies in which these adapted treatments are applied among DID patients indicated positive outcomes, with large effects on dissociative symptoms. The level of evidence for all currently available treatments for DID is still low, and in addition randomized controlled trials comparing effectiveness of the available treatments are lacking. As a result, it is not clear which intervention is most effective for treating DID. Therefore, an important next step for the near future is to systematically investigate the effectiveness of the aforementioned new applications in methodologically well-designed treatment studies, whereafter clinicians can be trained in treatment these methods. Hopefully, this will lead to broad, evidence-based diagnosis and treatment availability for this underserved patient group that goes beyond stabilization of symptoms.
